# Some Important Factors Which Influence Occlusion*Read before the National Dental Association, Milwaukee, Wisconsin, August 15-19, 1921. Reprinted in *Journal N. D. A.*, June, 1922.

**Published:** 1922-07

**Authors:** G. S. Monson


### SOME IMPORTANT FACTORS WHICH INFLUENCE OCCLUSION*

BY G. S. MONSON, D.D.S.

Whenever we find a man confining his efforts wholly or in part to full denture construction, we are bound to find a certain amount of grief and disappointment.

We have men working to perfect various technical details of our work, such as impressions, aesthetics, etc. As we perfect ourselves technically we gradually eliminate much of the grief just mentioned.

My research has convinced me that with these many details of technic must be combined reconstruction, keeping in mind anatomical relationships and physiological function.

Reconstruction on this basis with full dentures is bound to bring grief and disappointment in proportion to the operator's lack of knowledge of the anatomy and physiology of the parts involved. This knowledge must include the framework or osseous structure, origin and insertion of the major and depressor muscles of mastication, the structures of the throat and neck both superficial and deep.

Physiologically, we must know what is the normal action of the tributary organs to the mechanism of masti-

---

* Read before the National Dental Association, Milwaukee, Wisconsin, August 15-19, 1921. Reprinted in *Journal N. D. A.*, June, 1922.

cation. This includes the salivary glands, the tonsils, the tongue in the act of swallowing, the drainage, by swallowing, of the mouth, the posterior nares, and the Eustachian tubes. Finally, what makes the maximum efficiency of the air passages?

Our study of anatomy and physiology is with our subject in perfect health and no deformities. We are *them* ready to look for and recognize conditions which are evidence of pathology or at least deformities.

Let me at this time emphasize an important point with this question. "How can any man expect to prepare his patient mentally and carry her over the period of return to normal, without a thorough knowledge of the anatomy, physiology and pathology involved?"

It is only after careful study that the operator can reconstruct the lost facial dimension, which is merely the facial evidence of change in anatomical relationship. This in turn impairs functional action of the total organism, instead of as is commonly considered merely an impairment of the act of mastication. We are prone to accept as a test of efficiency for our dentures the lack of sore spots and the fact that our patient is satisfied with her efforts at mastication. We forget that our dentures should be a restorative prop as near like the original was when at its best as we can make it.

This restoration of facial dimension very often brings criticism from our patient, her family and friends, unless we educate them *all* by educating the patient to what we are trying to accomplish. *We* must know and make our patient understand that the change in the face which they criticize is but temporary.

When we use the term *restore* we are endeavoring to as nearly as possible place the mandible in its normal relation with the rest of the osseous structure of the face and cranium. This permits in a comparatively short time, depending on the amount of dimension lost, the integument and superficial structures of the face to assume the original position they were in before facial dimension was lost.

It is a fault with a great many operators that rather than go thru the trouble of educating the patient to wear dentures constructed with real restoration in mind, they will compromise for present appearance. Many times making no restoration of lost dimension whatever. It is better with a patient whose mental attitude is hard to influence, to construct a first set of dentures making a slight restoration until the patient can be won over; a series of dentures then with a little more restoration each time will in the end give the result desired.

In studying the subject of the construction of the human mechanism of mastication, it is a question whether to approach by a study of the bones forming the framework, or the study of the muscles which played such an important part in the development of those bones and all parts adjacent. A little thought will convince one that during the age of development, exercise of muscles creating stress on their origin and insertion must of necessity develop the bone to meet this stress. Even after the period of development the bones may be modified in form and relation due to nature's effort to compensate for abnormal stress, caused by muscular unbalance. This is evident when the relation of mandible to the base of the cranium and facial bones has been changed from its original by loss of teeth or improperly constructed dentures. Always bear in mind that the masticating apparatus was developed to its point of efficiency with natural teeth. Part or total loss of these natural teeth produces a condition which, if allowed to exist will not only give a change of relationship of mandible to the maxilla, but also a modification of the form of the bones. Further, the functions of mastication, deglutition, and respiration are all impaired in proportion to the amount of this changed anatomical relationship.

In discussing osseous anatomical relationship as regards the masticating apparatus, we must bear in mind that the only actual contact is in the glenoid fossae. A normal position in this relationship does not permit encroachment of the condyles on the external auditory meatus. This normal position is maintained by normal occlusion, because the only other contact of the mandible with the bones of the cranium is through the occlusion of the teeth. It is evident then that any change in occlusion due to wear on or loss of natural teeth or improperly constructed dentures will permit a change in the position of the condyles in the glenoid fossae. In thinking of occlusion, we are apt to confine our thoughts to the ability of the mandible to close up to the point of teeth in contact, giving little consideration to a tooth length which will restore the normal relationship of mandible to cranial and facial bones. In other words, occlusion is more than the ability of placing the teeth in contact in the act of mastication. A further result of normal relation of mandible to the rest of the osseous framework is the normal position of all structures of the face and neck which depend for this position upon the mandible.

Upon this erect a base of Bonwill triangle. Geometrically, we have determined the center of our base triangle. From the mesio-incisal angle, the center of the triangle and the line passing through the axis of the condyles, we project lines to similar points on the same skull in lateral view. The value of this diagrammatic figure in our present discussion comes from our projected center of applied force. A little study will convince one that with such a center to figure from it is easily demonstrated how great is changed osseous relationship because of shortened occlusion. This, of course, would be evidenced by a shortening of the line between the mesio-incisal angle and the center of applied force.

With this in mind, we arrive at the deduction that with this shortening of occlusion, producing a shorter line from mesio-incisal angle to center of applied force, we also will have a shorter distance between the origin and insertion of many of the major muscles of mastication.

Anatomy teaches that the term ''origin of masticating muscle'' is meant to imply its more fixed or central attachment, and the term ''insertion,'' the movable point to which the force of the muscle is directed. This is important because we should have a clear mental picture of muscular anatomy in order to thoroughly understand change of muscle tone which leads to unbalanced muscle energy.

Considering change of position or relation of the mandible, we have two principal groups of muscles to study. The great fan-shaped temporal radiating from its insertion in the coronoid process to its large spread out origin in the temporal bone, produces a pull upward and backward. Opposed to this, we have the group composed of the internal and external pterygoids and masseter. This group produces an action which is mainly upward and forward. These two forces, when all muscles involved are of equal or normal tone, gives what we term muscular balance controlling the main movements of the mandible.

The base of the skull shows the external pterygoid with its horizontal position from origin in the sphenoid to insertion in front of the neck of the condyle, producing the forward movement of the mandible as in exercising. The internal pterygoid with its direction from insertion in the lower and back border of the angle of the mandible to its origin in the pterygoid fossae of the sphenoid also near the tuberosity of the superior maxillary produces a pull upward and forward. Figure 4 is the masseter, with its origin in the zygoma and malar process and insertion in the greater part of the ramus having a main action to close the jaw but with considerable force directed forward. This last group we are most concerned with in our study of loss of muscle tone due to lost facial dimension. Any change of position causing the mandible to occupy a closer relationship to the maxilla than normal will naturally shorten the distance between the origin and insertion of the masseter and internal pterygoid. If we liken a muscle in its normal position as to its tone or muscle pull to a rubber band stretched to a certain position, we can readily see how the muscle will lose tone by lessening the distance between points, the same as the rubber band would by lessening the distance between the stretched ends. This loss of normal tone of these two muscles allows the horizontal fibers of the temporal to overbalance, and we have the first step in the backward movement of the mandible. As lost facial dimension progresses the temporal gaining in its overbalance, we get a shortening of distance between origin and insertion of external pterygoid as well, which condition when pronounced completes our vicious circle of deformity.

Conversely by opening the relationship of mandible to maxilla using as guide relief of condyle encroachment, we gradually put normal stretch back on these muscles. Soon muscular balance returns between groups and the mandible takes its normal position with condyles in their normal position in the glenoid fossae.

I contend that the normal position in a normal mouth, under good development is in occlusion. In opening these dentures and opening the mandible in relation to the maxilla there is tension, there is atrophy of those muscles, and we are not going to have any restoration until they are put back to their normal relation. There is always enough elasticity for movement of the muscle to give us a rest position, and in all development cases that I have had a chance to observe the natural jaws, the best development of the cranium and the mandibles were in relation to rest position. Interlocking of the cusps carries on the harmony of development of the two bones.

With regard to the functions that are impaired, let me cite an extreme case in my practice. A lady was sent to me by a nerve specialist. She could neither sleep nor rest. There was a roaring or slight buzzing in her ears. She was about thirty years of age. I opened the mandible in that case until I felt the impulse of the meatus, that is the anterior wall of the canal; it was three-eighths of an inch restoration. It was a painful experience to listen to her swallow. The hearing was improved immediately on putting in the dentures, by lifting the stress off the anterior wall of the canal, from nine inches to eighteen inches on one side, and from eleven inches to twenty-four inches on the other side. In a very short time she was swallowing like any normal individual; and there was a gain of twenty-four pounds; there was a better appearance, which is only a facial evidence of the mandible in its proper relation. In a short time there will probably be a space; in the event that she does not use those muscles and they lose their tone, there will be an inaction or dropping away of that occlusion.

